# Investigating the Different Mechanisms of Genotoxic and Non-Genotoxic Carcinogens by a Gene Set Analysis

**DOI:** 10.1371/journal.pone.0086700

**Published:** 2014-01-31

**Authors:** Won Jun Lee, Sang Cheol Kim, Seul Ji Lee, Jeongmi Lee, Jeong Hill Park, Kyung-Sang Yu, Johan Lim, Sung Won Kwon

**Affiliations:** 1 College of Pharmacy and Research Institute of Pharmaceutical Sciences, Seoul National University, Seoul, Republic of Korea; 2 Samsung Genome Institute, Samsung Medical Center, Seoul, Republic of Korea; 3 School of Pharmacy, Sungkyunkwan University, Suwon, Republic of Korea; 4 Seoul National University College of Medicine and Hospital, Seoul, Republic of Korea; 5 Department of Statistics, Seoul National University, Seoul, Republic of Korea; University of Hawaii Cancer Center, United States of America

## Abstract

Based on the process of carcinogenesis, carcinogens are classified as either genotoxic or non-genotoxic. In contrast to non-genotoxic carcinogens, many genotoxic carcinogens have been reported to cause tumor in carcinogenic bioassays in animals. Thus evaluating the genotoxicity potential of chemicals is important to discriminate genotoxic from non-genotoxic carcinogens for health care and pharmaceutical industry safety. Additionally, investigating the difference between the mechanisms of genotoxic and non-genotoxic carcinogens could provide the foundation for a mechanism-based classification for unknown compounds. In this study, we investigated the gene expression of HepG2 cells treated with genotoxic or non-genotoxic carcinogens and compared their mechanisms of action. To enhance our understanding of the differences in the mechanisms of genotoxic and non-genotoxic carcinogens, we implemented a gene set analysis using 12 compounds for the training set (12, 24, 48 h) and validated significant gene sets using 22 compounds for the test set (24, 48 h). For a direct biological translation, we conducted a gene set analysis using Globaltest and selected significant gene sets. To validate the results, training and test compounds were predicted by the significant gene sets using a prediction analysis for microarrays (PAM). Finally, we obtained 6 gene sets, including sets enriched for genes involved in the adherens junction, bladder cancer, p53 signaling pathway, pathways in cancer, peroxisome and RNA degradation. Among the 6 gene sets, the bladder cancer and p53 signaling pathway sets were significant at 12, 24 and 48 h. We also found that the DDB2, RRM2B and GADD45A, genes related to the repair and damage prevention of DNA, were consistently up-regulated for genotoxic carcinogens. Our results suggest that a gene set analysis could provide a robust tool in the investigation of the different mechanisms of genotoxic and non-genotoxic carcinogens and construct a more detailed understanding of the perturbation of significant pathways.

## Introduction

Based on their mechanisms of action, chemical carcinogens are classified as genotoxic carcinogens (GTXs) or non-genotoxic carcinogens (NGTXs). GTXs covalently bind with DNA to form DNA adducts, which results in neoplastic initiation [1]–[3]. In *in vitro* and short-term *in vivo* assays, GTXs have been observed to damage DNA and generate chromosomal aberrations [1], [2]. NGTXs, however, do not directly bind with DNA, instead, they cause neoplastic transformations through various mechanisms, including repression of the immune system and inducing oxidative stress [1], [2]. Therefore, it is hypothesized that GTXs and NGTXs induce distinct gene expressions profiles, which may consequently be used to classify unknown compounds as either GTXs or NGTXs [4]. Unlike NGTXs, many GTXs also cause tumor in animal-based carcinogenic bioassays and environment exposures to chemical carcinogens have been reported to be major causal factors for cancer [5]. From the perspective of health care safety and the pharmaceutical industry, determining the genotoxic potentials of chemicals to which humans are exposed is important to discriminate GTXs from NGTXs [5].

The potential genotoxicity of carcinogens is evaluated using *in vitro* tests, such as bacterial gene mutation test (Ames test), the mammalian micronuclei (MN) test, the chromosomal aberration (CA) test and the mouse lymphoma assay (MLA) [6]. To be classified as a genotoxic carcinogen, a chemical must exhibit *in vivo* genotoxicity in rodents. However, the *in vitro* results may not correspond with the results of *in vivo* evaluations, which result in numerous unnecessary animal experiments that are both costly and time consuming [7], [8]. Thus, a more robust *in vitro* method is required.

Toxicogenomics, the application of gene expression profiling to toxicological investigations, provides novel approaches to address this problem, leading to deeper mechanistic insights. These approaches have been demonstrated to discriminate between GTXs and NGTXs [1], [9]. To interpret gene expression profiling in a biologically meaningful way, individually identifying every gene with a statistically significant response is not sufficient [10]. Recently, the focus of studies has shifted from studying the effects of individual genes to studying the effects of a gene set, i.e., multiple functionally related genes [11], [12]. A few studies including on by Kim HS *et al.* [10] have demonstrated the successful application of gene set analysis using gene expression data. In this study, we conducted gene set analysis to discriminate between genotoxic and non-genotoxic mechanisms for the first time.

To apply a gene set analysis, we used 12 compounds as the training set (12, 24, 48 h) and validated significant gene sets using 22 compounds for the test set (24, 48 h). Using a cut-off of p<0.05 for at least 1 time point, we selected 57 significant gene sets from 5 GTXs and 7 NGTXs in the training data. To validate the 57 gene sets, we utilized the prediction analysis for microarrays (PAM) and the accuracy of each gene set was calculated using the 24 and 48 h time points in both the training and test data. Compared with previous studies, our results suggest that this method of applying gene set analysis could be used to more clearly explain the differences between GTX and NGTX mechanisms.

## Materials and Methods

### Data collection

Raw gene expression profiling data were obtained from the Gene Expression Omnibus through accession number GSE28878. In a microarray experiment, HepG2 cells were treated with GTXs or NGTXs. The HepG2 cell culture medium was replaced with fresh medium containing either compound or with the corresponding control. HepG2 cells were treated with the training set compounds for 12, 24 and 48 h and the test set compounds for 24 and 48 h [5].

The liver plays an important role in the metabolism of many compounds and represents a major target organ in systemic toxicity, therefore, hepatic models are frequently used among the *in vitro* models [13]. As a preferred model of hepatic cell lines, the human liver cell line (HepG2) is widely employed in studies on the biotransformation of xenobiotic compounds because it does not carry the p53 mutation and enables cells to induce the DNA damage response pathway, arrest growth and activate apoptosis [13]. Many studies have revealed that HepG2 cells are suitable and applicable for genotoxic assays including the MN test and the comet assay [14].

The genotoxicity of the carcinogens was evaluated using *in vitro* genotoxicity assays (MN, CA, MLA) and *in vivo* genotoxicity assays (MN, CA). Carcinogens were classified as GTXs when they caused positive results in the genotoxicity assays and NGTXs if they caused negative results [5]. To observe a clear difference between GTXs and NGTXs, we selected 16 GTXs that showed consistent genotoxicity in both the *in vitro* and *in vivo* assays and 18 NGTXs that showed consistent non-genotoxicity in both *in vitro* and *in vivo* assays in GSE28878.

Table 1 displays the details for each of the selected compounds. We used 12 compounds for the training set and 22 compounds for the validation set. The training data included 12, 24 and 48 h time points that were used for expression profiling, and the validation data included 24 and 48 h time points.

**Table 1:** Thirty-four compounds were classified as part of the training sets and test sets.

Dataset		Compound	Time (h)
Training	GTX	Aflatoxin B1	12,24,48
		Benzo[a]pyrene	12,24,48
		2-Acetyl aminofluorene	12,24,48
		Dimethyl nitrosamine	12,24,48
		Mitomycin C	12,24,48
	NGTX	2,3,7,8-Tetrachloro	12,24,48
		dibenzo-p-dioxin	12,24,48
		Wy 14643	12,24,48
		Cyclosporine A	12,24,48
		Ampicillin trihydrate	12,24,48
		Di(2-ethylhexyl) phthalate	12,24,48
		d-Mannitol	12,24,48
		Diclofenac	12,24,48
Test	GTX	Azathioprine	24, 48
		4-Aminobiphenyl	24, 48
		Benzidine	24, 48
		Chlorambucil	24, 48
		1-Ethyl-1-nitrosourea	24, 48
		4,4′-Methylenebis(2chloroaniline)	24, 48
		2-Amino-3-methylimidazo[4,5-f] quinolone	24, 48
		Cyclophosphamide	24, 48
		Cisplatin	24, 48
		Furan	24, 48
		Diethylnitrosamine	24, 48
	NGTX	Caprolactam	24, 48
		Coumaphos	24, 48
		Diazinon	24, 48
		Acesulfame-K	24, 48
		Progesterone	24, 48
		1,1,1-Trichloro-2,2-di-(4chlorophenyl) ethane	24, 48
		Lindane	24, 48
		Nitrobenzene	24, 48
		Simazine	24, 48
		Tetrachloroethylene	24, 48
		Pentachlorophenol	24, 48

Training sets included 5 GTXs and 7 NGTXs, and the test sets included 11 GTXs and 11 NGTXs. The time points were 12, 24 and 48 h of exposure for the training set and 24 and 48 h of exposure for the test set.

### Preprocessing

Human Genome U133 Plus 2.0 Gene Chip Arrays were used as the platform for the gene expression profile [5]. The data were normalized using a robust multi-array analysis (RMA) with the affy R package [15]. To convert the gene labels into Entrez IDs, we used the Database for Annotation, Visualization, and Integrated Discovery (DAVID) software [16]. At each time point, fold changes were calculated for each compound through a comparison to a corresponding control.

To remove batch effects, we used the ComBat method in the sva R package. The ComBat method can be applied to high dimensional data matrices using an empirical Bayesian framework, and the ComBat output is a corrected expression profile [17]. Our training and test datasets were processed for each of the 3 different days. We found that our expression profile had severe batch effects that were removed by the ComBat method (Figure S1).

### Selection and validation of significant gene sets

The aim of this gene set analysis was to search for gene set expression profiles related to GTXs or NGTXs [18]. We evaluated the differential gene expression patterns of gene sets derived from the Kyoto Encyclopedia of Genes and Genomes (KEGG) pathways and selected significant gene sets after exposure to 5 GTX and 7 NGTX.

For the gene set analysis, the Globaltest R package was used. Globaltest is a generalized linear model for predicting a response variable from the expression of gene sets [10], [18]. The null hypothesis of Globaltest is that there are no associations between the response (GTXs vs. NGTXs) and expression of the gene sets [18]. P-values were calculated from the “gt” function in Globaltest. We found 57 gene sets with a p<0.05 for at least one of the 12, 24 and 48 h time points. Additionally, using the “comparative” function in Globaltest, we calculated comparative p-values as false discovery rate (FDR) for multiple-comparisons of KEGG pathways.

To determine whether the 57 gene sets were significant, a prediction analysis for microarrays (PAM) was conducted. The PAM classifies samples from gene expression data using the nearest shrunken centroid method [19]. The nearest shrunken centroid classification is a modified standard nearest centroid classification. Using the nearest shrunken centroid, samples were classified by the subsets of genes that best characterize each class. PAM has been employed by numerous studies to predict class from gene expression data [20]–[23].

Using the fold changes of each of the 57 selected gene sets, PAM was performed to develop prediction models from the training set. Using the 57 prediction models, 12 training and 22 test compounds were predicted to classify into GTXs or NGTXs at 24 and 48 h, respectively. To generate a predictive model, a balanced 10-fold cross validation was conducted for each gene set. Using the PAM results, accuracy, sensitivity and specificity, were calculated. We selected the final 6 gene sets using an accuracy of > 90% for the training set and an accuracy of > 70% for the test set.

### Visualization

To visualize the 6 significant gene sets, we generated a gene plot using the Globaltest R package. The “Global Test Statistic” for each gene can be represented as the p-value from the component test in the Globaltest.

In the gene plot, we visualized the p-values of genes as bars. The gene with the lowest p-value contributed the most to the significance of the test result. The bars were colored to indicate a positive or a negative association of the gene expression with either GTXs or NGTXs. Thus, based on the comparison of GTXs with NGTXs, red bars indicate a gene that is up-regulated by a GTX and green bars indicate a gene that is down-regulated by a GTX. The threshold for statistical significance was set as p-value < 0.05. To further our understanding, we calculated the average fold change related to 5 GTXs and 7 NGTXs in training data and mapped the fold changes of individual gene to the KEGG pathway for each time point using pathview R package (http://bioconductor.org/packages/2.12/bioc/html/pathview.html). Pathview is used for data integration and visualization of pathways. This program maps a wide variety of biological data to a target pathway specified by user.

Principal component analysis (PCA) was performed using the R function, “prcomp” for the expression values of each of the 6 gene sets. Twelve compounds from the training data were distributed by 3D principal component analysis for each of the 3 time points.

To measure the classification performance, we used Kernel-based Orthogonal Projections to Latent Structures (K-OPLS) [24], [25]. Because the K-OPLS method has a unique ability to detect an unanticipated systemic variation, the results provide a robust model evaluation [24]. Additionally K-OPLS has been applied to model a variety of biological data [24], [25]. Using the K-OPLS R package, we implemented 100-permutations and calculated the area under the curve (AUC).

## Results and Discussion

### Gene set analysis and classification

**Figure 1:**
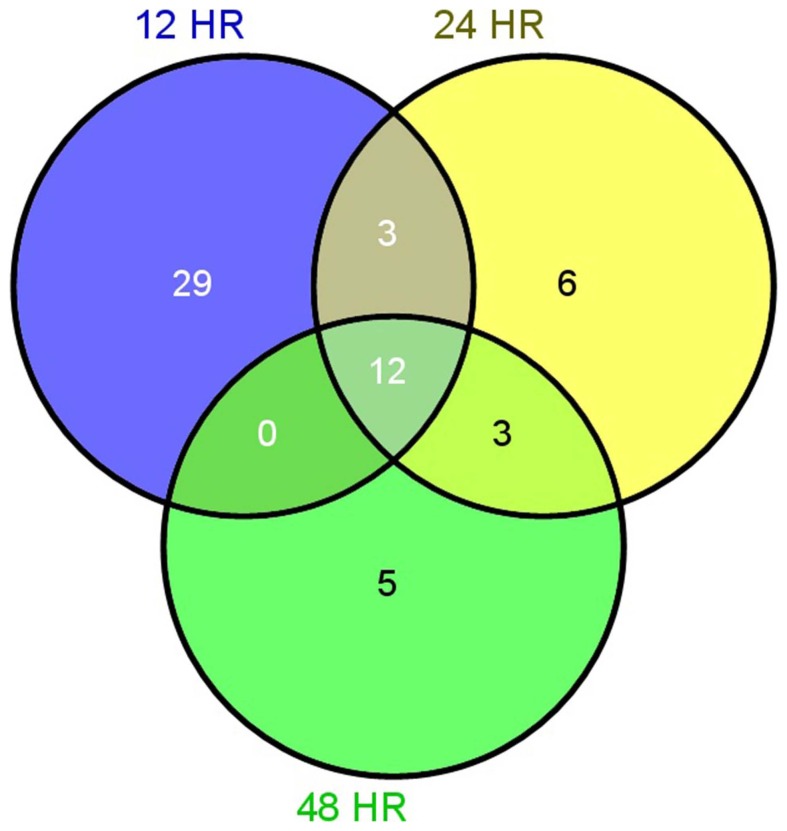
A Venn diagram displaying the 57 gene sets that met p < 0.05 for at least one of the 12, 24 or 48 h time points.

We conducted a gene set analysis to discriminate between GTXs and NGTXs and obtained a global test statistic. Because there were only a few gene sets that were significant at the p < 0.05 level, an unadjusted p-value below 0.05 was selected as the cut-off. There were 57 gene sets that satisfied p < 0.05 for at least one of the 12, 24 and 48 h time points (Table S1). We found that 12 gene sets were consistently activated at all 3 time points. The results also revealed that 29 gene sets were only activated at 12 h, 6 gene sets were activated only at 24 h and 5 gene sets were activated only at 48 h. We identified 3 gene sets that were activated both at 12 and 24 h but not 48 h. There were also 3 gene sets that were activated both at 12 and 48 h but not 24 h. We also found that 44, 24 and 20 gene sets were activated at 12, 24 and 48 h, respectively (Figure 1). This finding suggested that most gene sets were significantly activated at an earlier time point.

Using the 57 gene sets, 12 training and 22 test compounds were classified as GTXs or NGTXs and we determined the accuracy, sensitivity and specificity of the classification using PAM (Table S2). We calculated the accuracy, sensitivity and specificity as described in previous studies [26], [27]. In the classification results, a positive or negative value indicated that the compound was classified as a GTX or NGTX, respectively. Thus, a true positives (TP) is an actual GTX that was predicted to be a GTX, and a false positive (FP) is an actual NGTX that was predicted to be a GTX. Similarly, a false negative (FN) is an actual GTX that was classified as a NGTX, and a true negative (TN) is an actual NGTX that was predicted to be an NGTX. From the TP, FP, FN and TN rates, we calculated the accuracy, sensitivity and specificity of the 12 training and 22 test compounds.

Compared with the test dataset, the accuracy of each gene set was higher for the training dataset. We selected 6 gene sets with > 90% accuracy in the training set and > 70% accuracy in the test set (Table 2). These 6 gene sets included genes related to the adherens junction, bladder cancer, p53 signaling pathway, pathways in cancer, peroxisome and RNA degradation.

**Table 2:** To validate the 6 significant gene sets, PAM was conducted to classify the compounds using the fold changes of the 6 significant gene sets.

Gene Set Name	24 h						48 h					
	Training			Test			Training	Test
	accuracy (%)	sensitivity (%)	specificity (%)	accuracy (%)	sensitivity (%)	specificity (%)	accuracy (%)	sensitivity (%)	specificity (%)	accuracy (%)	sensitivity (%)	specificity (%)
Adherens junction	92	80	100	73	55	91	100	100	100	73	45	100
Bladder cancer	92	80	100	73	45	100	92	80	100	77	55	100
p53 signaling pathway	92	80	100	73	45	100	92	80	100	73	45	100
Pathways in cancer	100	100	100	73	45	100	100	100	100	73	45	100
Peroxisome	10	100	100	77	64	91	92	100	86	77	82	73
RNA degradation	92	80	100	73	45	100	100	100	100	73	45	100
Mean	95	87	100	74	50	97	96	93	98	74	53	96

Accuracy, sensitivity and specificity were calculated for both the training dataset and the test dataset.

Among the 6 gene sets, we found that the bladder cancer and p53 signaling pathway gene sets were significant for all 3 time points, the other gene sets were significant only at 12 h (Table 3). Even after correcting the p-value for the FDR, the bladder cancer and p53 signaling pathway gene sets were still significant at all 3 time points. According to the FDR, more gene sets were significantly activated at 12 h than at 24 and 48 h (Table 3). Because all 6 gene sets were significantly activated at 12 h, investigations of the gene expression at earlier time points would be beneficial. Such an investigation may explain why the bladder cancer and p53 signaling pathway gene sets were significant at all three time points, whereas other gene sets were significant only at the early time point (Table S1).

**Table 3:** P-values calculated from the Globaltest for each of the 3 time points in the training set.

Gene set Name	Training data		
	12 h	24 h	48 h
Adherens junction	0.035	0.111	0.338
Bladder cancer	0.010	0.008	0.015
p53 Signaling pathway	0.016	0.006	0.013
Pathways in cancer	0.039	0.056	0.277
Peroxisome	0.016	0.265	0.438
RNA degradation	0.008	0.205	0.207

The K-OPLS results indicated that 24 h of exposure to the training compounds resulted in a higher mean AUC than 48 h of exposure. Notably at 24 h, the p53 signaling pathway and bladder cancer gene sets exhibited robust performance with respect to classification, with an AUC of 0.907 and 0.861, respectively (Table 3).

### Gene plot and PCA analysis

To further evaluate the significant gene sets including p53 signaling pathway and bladder cancer pathway, we investigated time-dependent expression in gene plot. A gene plot explains the contribution of each individual gene in the significant test, and therefore, we were able to identify genes that were differentially expressed in the gene set. For GTX treated HepG2 cells, the gene plot indicated that significantly up-regulated genes were more dominant than down-regulated genes. In the bladder cancer gene set, TP53, RASSF1, CDKN1A and PGF were significantly up-regulated after 12 h of GTX exposure. At 24 h, MDM2, PGF, CDKN1A and E2F1 were significantly up-regulated by GTXs. PGF, MDM2 and CDKN1A were up-regulated by GTXs at 48 h (Figure S2).

In the p53 signaling pathway gene set, 13 genes (DDB2, EI24, PIDD, TP53, TP73, CDK2, PPM1D, SESN1, RRM2, CASP9, CDKN1A, APAF1, BAX) were significantly up-regulated by GTXs at 12 h (Figure S2). Five of these genes (PIDD, BAX, PIGs, APAF-1, CASP9) are known to be involved in apoptosis, and three of these genes (DDB2, SENS1, RRM2) are associated with DNA repair. TP75 and PPM1D are related to the negative feedback of p53.

**Figure 2:**
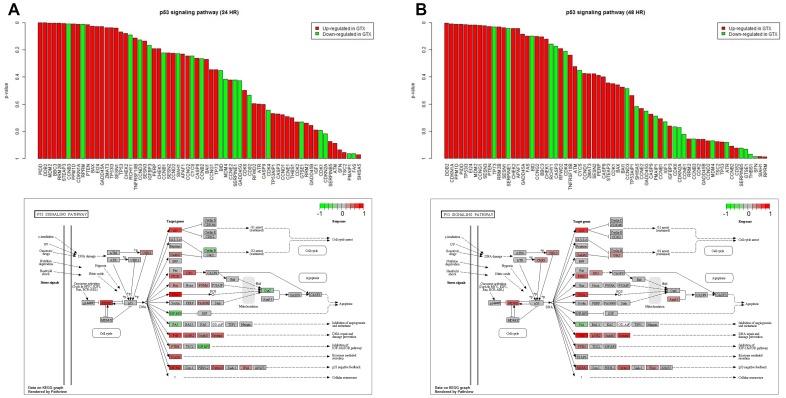
Gene plot (top) from Globaltest and KEGG pathway (bottom) showing the fold change of individual genes in the p53 signaling pathway. Red and green bars indicate up-regulated and down-regulated genes, respectively, after GTX exposure at A. 24 h or B. 48 h in comparison to NGTX exposure.

We visualized the p53 signaling pathway as gene plots for the 24 and 48 h points, shown in Figure 2a and Figure 2b, respectively. In the p53 signaling pathway, we found 17 and 13 significant (p < 0.05) genes at 24 and 48 h, respectively; the number of significant genes decreased as exposure time increased from 24 to 48 h. In Figure 2a, it can be observed that 17 genes were significant (PIDD, DDB2, MDM2, BBC3, RRM2B, STEAP3, CCNB3, PPM1D, CDKN1A, RPRM, PTEN, BAX, EI24, GADD45A, ZMAT3, TP53I3, SESN1). In Figure 2b, it can be observed that 13 genes were significant (DDB2, CDKN1A, PPM1D, PIDD, TP53I3, EI24, MDM2, CCNG1, SESN3, PTEN, TP73, RRM2B, and SESN1).

We compared the significant genes in each functional group to understand the functional changes in the p53 signaling pathway. Figure 2a shows that four genes (PIDD, BBC3, BAX, EI24) were involved in apoptosis at 24 h, and three genes (DDB2, RRM2B, GADD45A) were associated with DNA repair at 24 h. MDM2 and PPM1D were related to the negative feedback of p53. Figure 2b shows that three genes (PIDD, TP53I3, EI24) were involved in apoptosis at 48 h, and three genes (DDB2, SESN3, RRM2B) were associated with DNA repair. MDM2, CCNG1 and PPM1D were related to the negative feedback of p53.

In both Figure 2a and Figure 2b, it can be observed that the identical number of DNA repair-related genes were consistently up-regulated; however, the number of apoptosis-related genes decreased from four (Figure 2a) to three (Figure 2b). The number of p53 negative feedback-related genes increased from two (Figure 2a) to three (Figure 2b).

The KEGG pathway and the fold-changes of individual genes, presented in the bottom of Figure 2, showed that several apoptosis-related genes (shown in Figure 2a) were up-regulated, but these up-regulated genes lost their expressions (Figure 2b). However, the fold-changes of the DNA repair-related genes shown in Figure 2a and Figure 2b were consistent.

By increasing the exposure time from 12 to 48 h, the number of significantly up-regulated genes related to apoptosis decreased from five to three, whereas the same number of DNA repair-related genes was consistently up-regulated for the GTX-treated HepG2 cells. Notably, TP53 is known to be involved in the suppression of tumors and was significantly up-regulated at 12 h; this significance was lost at 24 and 48 h. Instead, MDM2, a known negative regulator of the p53 tumor suppressor, was significantly up-regulated at 24 and 48 h.

At all 3 time points, the DNA damage-binding protein 2 (DDB2) was highly up-regulated in GTX- treated HepG2 cells. A recent study suggested that p53-triggered up-regulation of DDB2 is associated with a resistance to cell death that is induced by melanoma therapy in malignant melanoma cells [28]. Compared with the 12 h time point, the number of significantly up-regulated genes related to the negative feedback of p53 was increased at 48 h.

**Figure 3:**
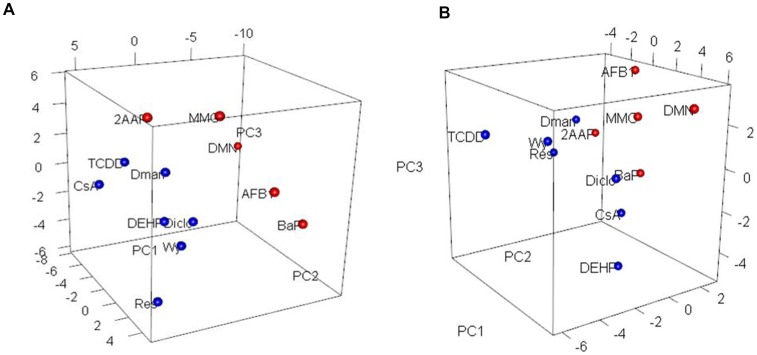
Principal component analysis revealed the distribution of 12 compounds in the training data. A. PCA results for gene expression in the p53 signaling pathway gene set at 24 h [red, 5 GTX; blue, 7 NGTX]. B. PCA results for gene expression in the bladder cancer gene set at 48 h [red, 5 GTX; blue, 7 NGTX].

A PCA analysis revealed that the 12 compounds in the training set were appropriately classified into either GTXs or NGTXs for both the bladder cancer gene set and p53 signaling pathway gene set, particularly at 24 h (Figure 3). Additionally, 34 compounds in the training and test data were separated by the expression of the p53 signaling pathway and bladder cancer gene sets at 24 h (Figure S3).

## Conclusions

To identify the differences between the GTX and NGTX biological mechanisms, we conducted a gene set analysis and validated significant gene sets. In previous studies, each gene was individually identified and classified using only statistical processes, and each of the individual classifiers was unrelated to biological mechanisms. However, information regarding biological processes is available for each gene in our study; thus, our method offers a simplified approach for explaining the different mechanisms of GTXs and NGTXs.

In a previous study, Magkoufopoulou *et al.* [5] suggested that their reported classifiers had a high classification accuracy at 24 h. Because they selected their classifiers from Ames-positive and Ames-negative compounds separately, the classifiers could be associated with different genotoxic properties. They also validated Ames-positive and Ames-negative compounds separately. This means that their classifiers may be limited in that they can only integrate information regarding Ames-positive and Ames-negative compounds. To evaluate genotoxicity using both *in vivo* results and Ames test results, we conducted gene sets analysis using 16 GTX that showed consistent genotoxicity in both the *in vitro* and *in vivo* assays and 18 NGTX that showed consistent non-genotoxicity in both *in vitro* and *in vivo* assays. The findings indicated that our gene sets could explain the genotoxic mechanism using both *in vivo* and Ames tests.

Our results revealed that at the 3 different time points, the expression of most gene sets was significantly activated at 12 h. Therefore, even if the previous study obtained their classifiers and validated them at 24 h, the expressions of genes at 12 h could provide more information on the mechanism of genotoxicity.

Although we identified gene sets that could discriminate between the GTXs and NGTXs biological processes, these gene sets could not explain why the compounds showed different results for the *in vivo* and *in vitro* assays. Additionally, in validation, the accuracy of test compounds was not as good as the training data. In conclusion, by employing gene set analysis, we found that the p53 signaling pathway and bladder cancer gene sets most accurately discriminated between GTXs and NGTXs. Additionally, our results suggested that gene expression at the early time point could provide more information regarding the initiation of carcinogenesis than that at a later time point. We further concluded that significantly expressed genes are involved in DNA repair, apoptosis and the negative feedback of p53.

## Supporting Information

Figure S1 **A.** Clustering of the 12 h training data, which was influenced by 3 different groups [a, Series A; b, Series B; c, Series C]. **B.** After applying the ComBat method, the output revealed that batch effects from the different groups were removed.
(TIF)

Figure S2 **Gene plot from Globaltest showed time-dependent expression of bladder cancer gene set.** **A.**12 h, **B.** 24 h, **C.** 48 h, **D.** Gene plot from Globaltest showing the p53 signaling pathway gene set at 12 h.
(TIF)

Figure S3 **Thirty-four compounds including training and test data were separated using PCA. A.** The expression of p53 signaling pathway was used in PCA at 24 h. **B.** The expression of bladder cancer was used in PCA at 24 h.
(TIF)

Table S1 **Globaltest statistic of the 57 gene sets that satisfied p < 0.05 for at least one of the 12, 24 and 48 h time points.**
(XLSX)

Table S2 **Using the 57 gene sets, 12 training and 22 test compounds were classified as GTXs or NGTXs and accuracy, sensitivity and specificity were obtained from the results of classification using PAM.**
(XLSX)

Table S3 **In training dataset, FDR were calculated by function “comparative” of Globaltest.** AUC generated by K-OPLS for measuring the performance of classification.
(XLSX)
