# Supplementary figures and images for: Investigating the Different Mechanisms of Genotoxic and Non-Genotoxic Carcinogens by a Gene Set Analysis
Source: PLoS One. 2014 Jan 31;9(1):e86700. doi: 10.1371/journal.pone.0086700 (PMC3908933; doi:10.1371/journal.pone.0086700)

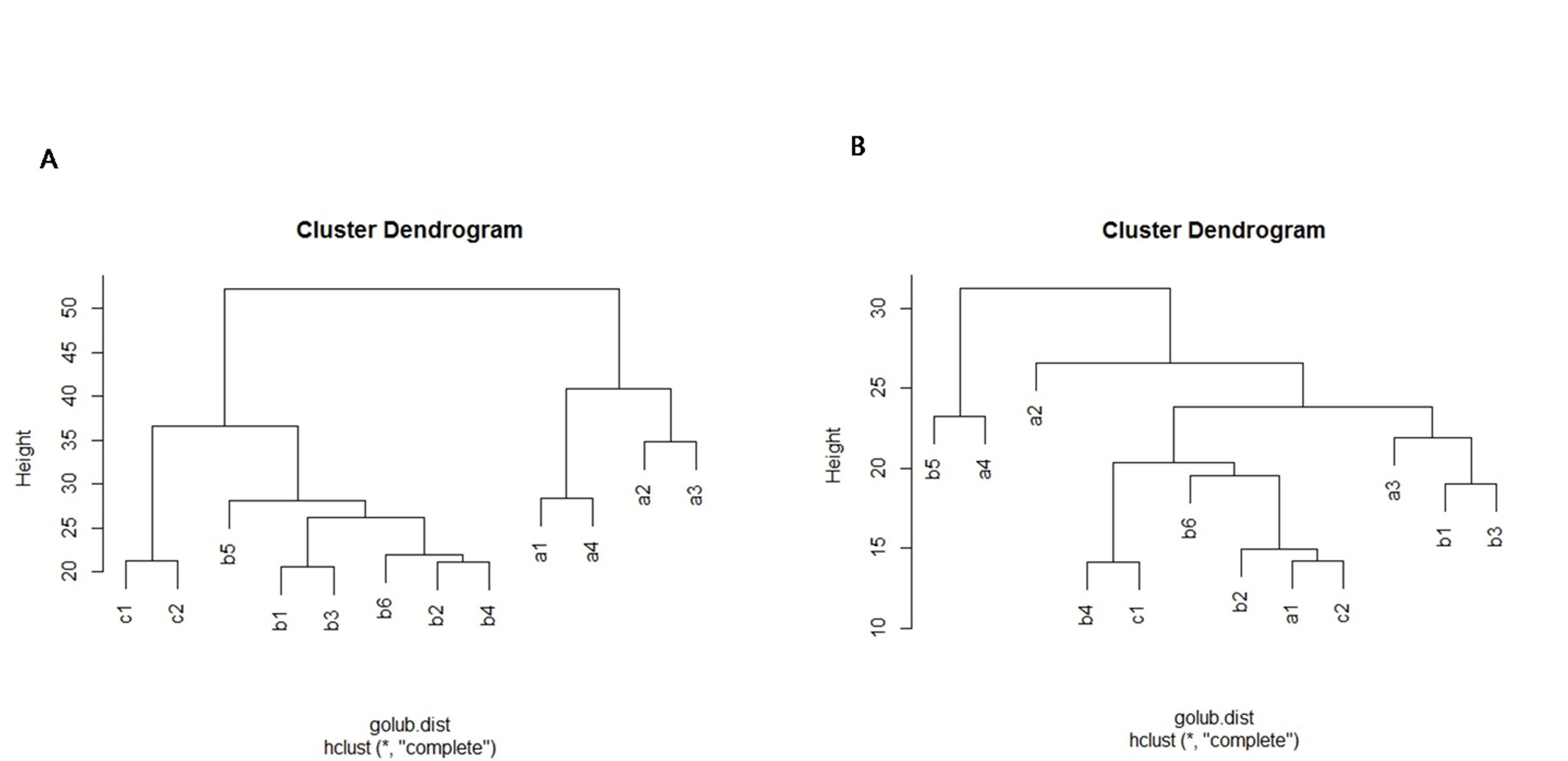

Supplement: Figure S1 — A. Clustering of the 12 h training data, which was influenced by 3 different groups [a, Series A; b, Series B; c, Series C]. B. After applying the ComBat method, the output revealed that batch effects from the different groups were removed. (TIF) [file pone.0086700.s001.tif]

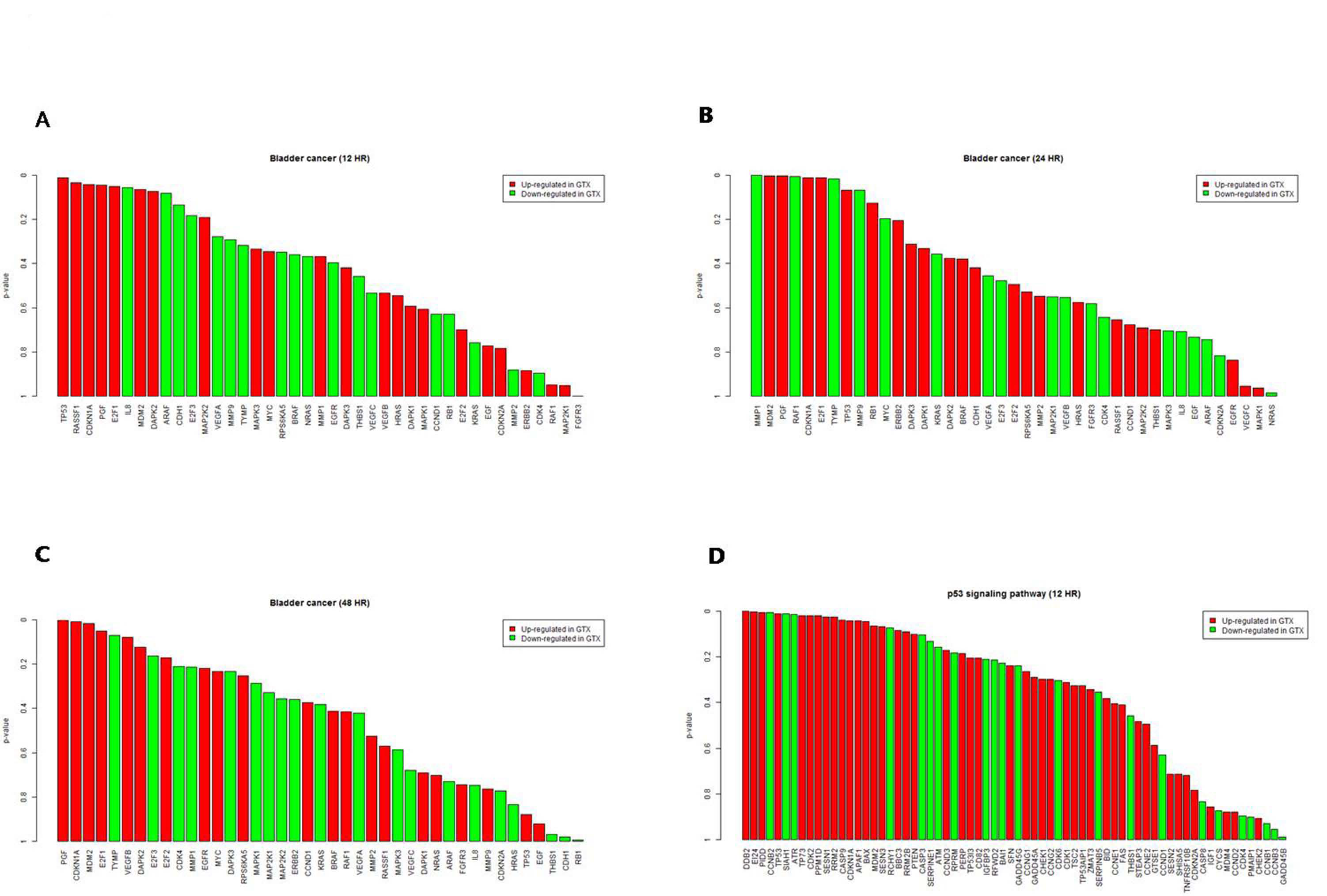

Supplement: Figure S2 — Gene plot from Globaltest showed time-dependent expression of bladder cancer gene set. A.12 h, B. 24 h, C. 48 h, D. Gene plot from Globaltest showing the p53 signaling pathway gene set at 12 h. (TIF) [file pone.0086700.s002.tif]

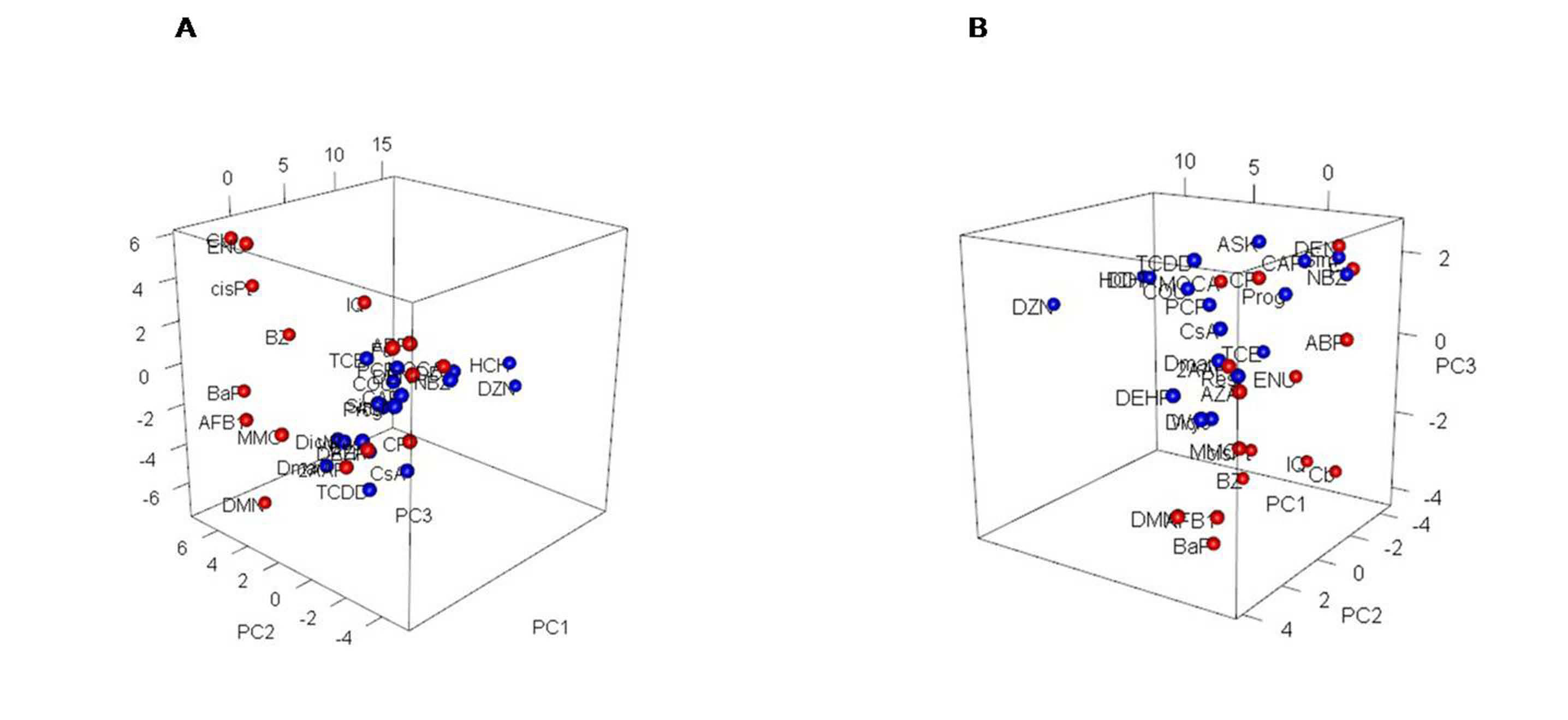

Supplement: Figure S3 — Thirty-four compounds including training and test data were separated using PCA. A. The expression of p53 signaling pathway was used in PCA at 24 h. B. The expression of bladder cancer was used in PCA at 24 h. (TIF) [file pone.0086700.s003.tif]
